# Hypothyroidism in the older population

**DOI:** 10.1186/s13044-019-0063-3

**Published:** 2019-02-08

**Authors:** Owain Leng, Salman Razvi

**Affiliations:** 1 Department of Endocrinology, Newcastle upon Tyne Hospitals NHS Foundation Trust, Newcastle upon Tyne, NE1 4LP UK; 2 Department of Endocrinology, Gateshead Health NHS Foundation Trust, Queen Elizabeth Hospital, Gateshead, Gateshead, NE9 6SX UK; 3 Institute of Genetic Medicine, Newcastle University, Newcastle upon Tyne, NE1 3BZ UK

**Keywords:** Hypothyroidism, Elderly, Ageing, TSH

## Abstract

**Background:**

Both overt hypothyroidism as well as minor elevations of serum thyrotropin (TSH) levels associated with thyroid hormones within their respective reference ranges (termed subclinical hypothyroidism) are relatively common in older individuals. There is growing evidence that treatment of subclinical hypothyroidism may not be beneficial, particularly in an older person. These findings are relevant at a time when treatment with thyroid hormones is increasing and more than 10–15% of people aged over 80 years are prescribed levothyroxine replacement therapy.

**Main body:**

The prevalence of hypothyroidism increases with age. However, the reference range for TSH also rises with age, as the population distribution of TSH concentration progressively rises with age. Furthermore, there is evidence to suggest that minor TSH elevations are not associated with important outcomes such as impaired quality of life, symptoms, cognition, cardiovascular events and mortality in older individuals. There is also evidence that treatment of mild subclinical hypothyroidism may not benefit quality of life and/or symptoms in older people. It is unknown whether treatment targets should be reset depending on the age of the patient. It is likely that some older patients with non-specific symptoms and incidental mild subclinical hypothyroidism may be treated with thyroid hormones and could potentially be harmed as a result. This article reviews the current literature pertaining to hypothyroidism with a special emphasis on the older individual and assesses the risk/benefit impact of contemporary management on outcomes in this age group.

**Conclusions:**

Current evidence suggests that threshold for treating mild subclinical hypothyroidism in older people should be high. It is reasonable to aim for a higher TSH target in treated older hypothyroid patients as their thyroid hormone requirements may be lower. In addition, age-appropriate TSH reference ranges should be considered in the diagnostic pathway of identifying individuals at risk of developing hypothyroidism. Appropriately designed and powered randomised controlled trials are required to confirm risk/benefit of treatment of subclinical hypothyroidism in older people. Until the results of such RCTs are available to guide clinical management international guidelines should be followed that advocate a conservative policy in the management of mild subclinical hypothyroidism in older individuals.

## Background

The population of the world is ageing. In the United Kingdom, nearly one in seven people is projected to be aged over 75 years by the year 2040. [1] However, increases in health life expectancy measured at 65 and 85 are not keeping pace with improvements in numerical life expectancy. This suggests that real health improvements are being experienced by younger people, and that people over 65 years of age are spending more time in ill-health. Therefore, unless this trend can be reversed, a major challenge for an ageing population is likely to be an increasing prevalence of the health conditions associated with old age such as dementia, type 2 diabetes mellitus and cardiac diseases. Apart from the effects on individuals and their families, this demographic change will have major socioeconomic and political implications.

Thyroid hormones have a major influence on all major organs/systems and adequate levels are important for optimal function. Thyroid dysfunction is a common condition that affects between 3 and 21% of the population with prevalence being more common in women and in older individuals. [2] In the UK, it is estimated that hypothyroidism treated with levothyroxine may affect nearly 800,000 older individuals aged more than 70 years. [3] The clinical presentation of thyroid dysfunction is non-specific and often variable; therefore, the diagnosis of thyroid dysfunction is based primarily on biochemical abnormalities. The pituitary hormone thyrotropin (TSH) has a complex inverse relationship with the thyroid hormones thyroxine (T4) and tri-iodothyronine (T3). A negative feedback mechanism exists between TSH and thyroid hormones, which means that TSH levels are the most sensitive marker of thyroid status in an individual. [4] Accordingly, overt hypothyroidism is defined as serum TSH concentrations above the reference range with low free T4 levels, while subclinical hypothyroidism is diagnosed when TSH levels are high and circulating free T4 is normal. The relationship between TSH and thyroid hormones is influenced by a number of factors including age, smoking and thyroid peroxidase antibody status. [5] Recent data from observational studies suggest that serum TSH levels increase in older people. [6] Thus, very mild TSH elevations in older individuals may not reflect subclinical thyroid dysfunction but rather be a normal consequence of ageing. Besides, serum TSH levels are also influenced by genetic, environmental, clinical and therapeutic factors and agents, as well as trends in clinical practice [7]. Despite this, adult patients are often managed similarly utilising a uniform serum TSH reference range (usually 0.4–4.5 mU/L) and age-specific ranges are not in routine clinical use. In addition, thyroid hormone requirements change with age and older patients on replacement therapy are more susceptible to the effects of thyroid hormone excess such as osteoporosis and atrial fibrillation. Therefore, careful consideration is required in the interpretation of thyroid function test results as well as in managing thyroid disease in the older population. The interest in thyroid function in the elderly has been increasing with the recognition that thyroid status is may be linked to disability, cognitive function, cardiovascular disease risk and longevity.

This review describes the prevalence of hypothyroidism in the older population and outlines the effects of treatment in this age group.

## Main text

### Prevalence of hypothyroidism in the elderly

Hypothyroidism is more prevalent in older individuals. The Whickham survey was the first population-based study to evaluate the presence of thyroid dysfunction in community-dwelling individuals. This seminal study observed that TSH levels increased with age in women after the age of 45 years but the same phenomenon was not seen in men. [8] The main limitation of the Whickham study, however, was that it utilised the first-generation TSH assay available at that time and was therefore unable to reliably detect TSH levels lower than 1.0 mU/L. Subsequently, a number of cross-sectional studies have been performed across various geographical locations and studying various age groups. In the studies restricted to older persons, the reported prevalence of overt hypothyroidism has ranged between 0.2–5.7% and subclinical hypothyroidism between 1.5–12.5%. [9–20] Some of the main prevalence studies are outlined in Table 1. The wide variation between the various studies probably reflects the disparate nature of the populations being assessed with regards to their gender, iodine intake, age-groups, racial groups and treated thyroid disease prevalence. For example, the Zoetermeer study from the Netherlands reported the lowest prevalence of subclinical hypothyroidism of just 1.5%, most likely due to the inclusion of only men in this analysis. [19]

**Table 1:** Prevalence of hypothyroidism (both overt and subclinical) in older population-based cross-sectional studies

Study [reference]	Place	Sample size	Population studied	Age range (years)	Measurement of thyroid function	Prevalence (%) Overt Subclinical Hypothyroidism
Framingham [9]	USA	2139	Both sexes	> 60	TSH & T4	2.5 7.9
Rotterdam [10]	Netherlands	10,318	Both sexes	≥ 45	TSH & FT4	0.8 9.1
Nagasaki [13]	Japan	2550	Atomic bomb survivors of both sexes	58.5^*^	TSH & FT4	NR 10.1
Cardiovascular Health Study [14]	USA	3233	Both sexes	> 65	TSH & FT4	1.6 15.0
Health ABC [15]	USA	2730	Both sexes	70–79	TSH & FT4	0.8 12.4
Zoetermeer [19]	Netherlands	403	Men only	73–94	TSH, FT4, FT3, rT3	0.2 1.5
Leiden 85+ [16]	Netherlands	558	Both sexes	85	TSH & FT4	7 5.0
Birmingham [17]	England	5960	Both sexes	≥ 65	TSH & FT4	0.4 2.9
Sau Paulo Ageing and Health Study [11]	Brazil	1373	Both sexes	≥ 65	TSH, FT4	5.7 6.5
Newcastle 85+ [18]	England	643	Both sexes	85	TSH, FT4, FT3, rT3	0.9 12.5
Longitudinal Aging Study [20]	Netherlands	1219	Both sexes	≥ 65	TSH	NR 5.3
InChianti study [23]	Italy	951	Both sexes	≥ 65	TSH, FT4, FT3	0.5 3.0

^*^Mean age provided as minimum age not available

As the TSH distribution and the reference limits shift to higher concentrations with age, the prevalence of subclinical hypothyroidism may be overestimated. Employing a uniform TSH reference range across all age groups in the NHANES study led to approximately 70% of older individuals with a slightly high serum TSH being incorrectly classed as having subclinical hypothyroidism. [21] An analysis of TSH results from one pathology centre in Western Australia however concluded that the use of age-specific TSH reference ranges has minimal impact on reclassifying thyroid status except in the very old (85 years), in whom 2–4.7% were reclassified as being euthyroid. [22] The reference ranges for TSH are discussed in the next section in more detail.

### TSH reference range in the elderly

Biochemical testing of thyroid function is fundamental to establish a diagnosis of thyroid dysfunction including hypothyroidism. The tests include measurement of circulating TSH and thyroid hormones in the serum. Assays for estimating serum TSH have improved vastly over the last few decades and the current immunoassays have the ability to detect very low levels (less than 0.1 mU/L). On the other hand, the reference range for thyroid hormones is wide for a given population, therefore, in principle, TSH will be the first detected circulating abnormality as the pituitary registers that T4 has changed from its genetically determined setpoint for that particular individual. [24] Thus, TSH measurement has now been firmly established as the first-line thyroid function test to assess thyroid status in the vast majority of patients with suspected thyroid disease. [25, 26] However, it is important to remember that measurement of serum TSH is only reliable for diagnosing thyroid function abnormalities provided that patients are not receiving drug therapies that alter TSH secretion or have pituitary disease. Measurement of serum TSH is also considered to be the key thyroid function test for diagnosing early (also called mild or subclinical) hypo- or hyperthyroidism because of the log-linear relationship between TSH and T4: a twofold change in serum FT4 level leads to a 100-fold alteration in circulating TSH. [27]

The American National Academy of Clinical Biochemistry formulated guidelines in 2003 which state that “TSH reference intervals should be established from the 95% confidence limits of the log-transformed values of at least 120 rigorously screened normal euthyroid volunteers who have: (a) No detectable thyroid autoantibodies, TPOAb or TgAb (measured by sensitive immunoassay); (b) No personal or family history of thyroid dysfunction; (c) No visible or palpable goitre and, (c) Who are taking no medications except oestrogen”. [28] In addition, TSH secretion has a diurnal variation with a peak late at night/early hours of morning, and, therefore, sample timing and shift work should also be considered when defining the TSH reference range. [29] In the last few decades, the ability of the TSH assays to detect lower levels has improved with each generation and therefore the present lower euthyroid reference limit is set at 0.3–0.5 mU/L. This has resulted in subclinical hyperthyroidism being diagnosed with much greater precision, irrespective of the population being studied or the method used. In contrast, the upper (97.5 percentile) reference limit for nonpregnant adults is still not universally agreed. [30, 31] As a consequence, the diagnosis of subclinical hypothyroidism is still very much dependent on the value at which the upper limit of TSH is set.

The TSH reference range should also consider the intra-individual variability of the TSH measurement. Several studies provide data showing significant variation in repeated TSH measurements over time in the same individuals. [27] Each person has a specific and unique setpoint for thyroid hormone concentrations, which is partly genetically determined, as shown by twin studies. [24] TSH measurements in an individual vary within 50% of the entire group’s TSH distribution, with a large and clinically significant variation. [32] TSH levels are also known to increase with age when checked over many years. In both the Cardiovascular Health Study as well as the Busselton Health study, there was a significant rise in TSH levels with little or no change in FT4 levels over a 13-year period. [33, 34] This finding, however, was not confirmed in the Rotterdam study in which TSH levels remained stable over a 6.5-year interval whereas FT4 levels increased. [10] An analysis from the Baltimore Longitudinal Study of Aging has revealed that changes in thyroid function tests are common, especially in older age groups, and regression to the mean is partly responsible for this finding. Importantly, changes in both TSH and FT4 over a 7-year period were associated with increased mortality. [35]

**Fig. 1:**
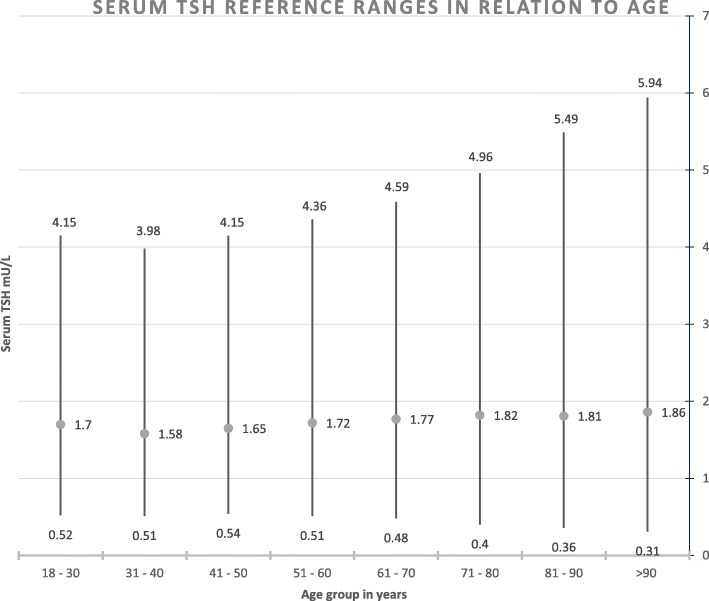
Distribution of serum Thyrotropin (TSH) in relation to age. The lower and upper limits of the TSH reference range are calculated from the 2.5th and 97.5th percentile, respectively, and the circle signifies the median value. (adapted from reference [37])

The most robust data determining the TSH reference range was obtained from the US National Health and Nutritional Examination Survey (NHANES) III study. [36] This large study (*n* = 16,088), designed to be representative of the US general population, analysed the median and lower and upper reference limits of serum TSH in carefully selected euthyroid individuals using current immunoassays. This study concluded that establishing an accurate TSH upper limit at an individual level from population data is not possible, as TSH has a low individuality index (the ratio between the within- and between-person variability). The overall reference range was deemed to be 0.4–4.1 U/L but there were significant differences between age groups and races. For example, the upper limit of TSH was 3.5 mU/L in the 20–29-year olds but increased to 7.9 mU/L in the 80+ year group. Similarly, the upper TSH level was 4.2 mU/L in White people whereas it was 3.4 mU/L in Black people. Similar data obtained from a Scottish laboratory database confirms an age-related increase in the upper reference limit for serum TSH). [37] An illustration of age-specific TSH reference ranges are described in the Fig. 1.

Serum TSH is not normally distributed and has a skew to the right. However, more than 95% of healthy euthyroid individuals have serum TSH values between 0.4 and 2.5 mU/L. It is therefore argued that TSH values > 2.5 mU/L reflect underlying autoimmune thyroid disease and contribute to the skewed TSH distribution curve, [38] a view further supported by the fact that such individuals have a higher risk of progression to subsequent hypothyroidism. [39, 40] The opposing argument to retain the upper limit of the TSH reference range around the 4.0–5.0 mU/L mark is that reducing the upper TSH reference limit would lead to a vast increase in the number of people diagnosed with subclinical hypothyroidism without any evidence-based justification or proof of benefits of treatment. [31] This issue is complicated by the concern that current TSH immunoassays differ in specificity for recognizing circulating TSH isoforms and that this can give rise to a full 1.0 mU/L difference in TSH values reported by different assays.

In summary, the current upper limit of the serum TSH reference range in older people does not reflect age-related changes and leads to the over-diagnosis of hypothyroidism and, consequentially, the probable unnecessary treatment of an unknown number of people with thyroid hormones. The adoption of a universal TSH range across all adult age groups on an individual’s health have not been tested in prospective trials, and unnecessary treatment will lead to a higher health and economic burden. In addition, a slightly higher serum TSH level may be normal in older individuals and not associated with worse outcomes. [18, 33] This has implications for diagnosing subclinical hypothyroidism in the elderly and also the level of serum TSH to aim for in treated hypothyroid patients in this age group. [41] Therefore, it has been suggested that age-specific reference limits should be utilised instead. [42] However, further research is required before age-specific TSH reference ranges become part of routine clinical practice.

### Consequences of overt and subclinical hypothyroidism in the elderly

#### Symptoms

The presentation of overt hypothyroidism in the older person is varied, non-specific, and often insidious. The classic symptoms of hypothyroidism are less likely to be evident in the elderly population, and if present, these symptoms are more likely to be misattributed to either co-morbid conditions or a manifestation of the ageing process. [43] Older people with hypothyroidism report fewer symptoms compared to younger counterparts. [44, 45] A prospective study comparing the frequency of reported symptoms has indicated that hypothyroid patients ≥70 years are significantly less likely to report weight gain, muscle cramps or cold intolerance than hypothyroid patients < 50 years of age. [45] A study comparing patients with overt autoimmune hypothyroidism against matched euthyroid controls found that whilst younger patients were more likely to report all 13 of the surveyed symptoms of hypothyroidism than their euthyroid controls, for older patients only three of the thirteen symptoms (tiredness, shortness of breath, wheezing) were more prevalent in the hypothyroid group than in the control group. The study used receiver operating characteristic (ROC) analyses to assess the discriminatory ability of a symptom score in the prediction of hypothyroidism, and whilst they identified that this was an excellent tool for predicting hypothyroidism in young men (with an area under the ROC curve of 91%; 95% CI 82–99.8%), it was poor in evaluating older women (area under the ROC curve of 64%; 95% CI 54–75%). [44] The non-specific and subtle presentation of hypothyroidism in older people is further evidenced by the studies into the screening for hypothyroidism in the older population, which have shown only a minority of patients with confirmed biochemical overt hypothyroidism have symptoms suggestive of the disease. [46, 47]

Whilst it is apparent that for most older patients the presentation of hypothyroidism is both more subtle and less discriminatory than in younger populations, there is however also an increased risk of the most severe presentation of hypothyroidism in the population: myxoedema coma. [48] Manifesting with multisystem failure with clinical features including reduced consciousness, hypothermia, hypotension, bradycardia, hyponatremia, hypoglycaemia, and hypoventilation, this is a condition with very high morbidity and mortality. However, myxoedema coma is a rare manifestation of hypothyroidism: an analysis of a Japanese national inpatient database estimated an annual incidence of 1.08 per million population. [48] Symptoms are largely absent or very subtle in older patients with subclinical hypothyroidism. In the subclinical hypothyroid group as a whole, most patients are asymptomatic or report only non-specific symptoms. [49, 50]

Thus, symptoms of hypothyroidism are sparse and non-specific in older people. This leads to thyroid function tests being frequently requested. However, due to the uniform TSH reference range being applied across all age groups, a substantial number of individuals are being detected with mild subclinical hypothyroidism. The median level of TSH at which treatment with thyroid hormones is being commenced has been falling recently although the evidence of benefit is sparse. [51]

#### Cardiovascular manifestations

The cardiovascular system is a major target of thyroid hormone action and sensitive to small changes in thyroid hormone concentrations. [52] A number of observational studies have suggested that even slight reductions in thyroid hormones are associated with higher risk of cardiovascular disease. [53]

Meta-analyses of observational studies have shown that subclinical hypothyroidism is related to an increased risk of ischaemic heart disease only in younger individuals and not in older populations. [54, 55] However, an individual patient meta-analysis of more than 55,000 participants contributing more than 500,000 person-years of follow-up concluded that age does not influence the relationship between subclinical hypothyroidism and coronary heart disease events nor mortality. [15]

Thyroid hormones have an inotropic effect on cardiac muscle. [56] Accordingly, some studies have shown a positive association between hypothyroidism and heart failure. [15] An individual participant data meta-analysis of 25,390 participants revealed that both low as well as high serum TSH levels. [57] In stratified analysis, there was a trend towards lower risk of heart failure in older individuals with subclinical hypothyroidism although this did not reach statistical significance.

The relationship between hypothyroidism and stroke has not been completely elucidated. A meta-analysis of individual participant data obtained from nearly half a million person-years of follow up demonstrated no overall effect of subclinical hypothyroidism on stroke although a significantly higher risk was observed in participants younger than 65 years and those with higher serum TSH levels. [58]

In a longitudinal analysis of participants from the Rotterdam study, the risk of sudden cardiac death was found to be higher with higher FT4 levels, even within the reference range. [10] In age-stratified analysis, the risk of sudden cardiac death appeared to be particularly higher in older individuals (> 65 years) with higher FT4 levels or lower TSH concentrations.

Older individuals (> 65 years) with subclinical or overt hypothyroidism were not observed to have adverse cardiovascular risk factors such as higher body mass index, increased LDL cholesterol, and prevalence of hypertension or diabetes mellitus in the Cardiovascular Health Study. [14]

#### Cognition

The relationship between overt hypothyroidism and cognitive impairment, depression and other psychiatric manifestations has been long-considered. The term ‘myxoedema madness’ was coined to describe the constellation of confusion, disorientation and psychosis that was observed to occasionally accompany profound hypothyroidism. [59] These early observations were later supplemented by physiological studies which showed alterations in electroencephalograms, cerebral blood flow, and visual evoked potentials in hypothyroid patients. [60, 61] Hypothyroidism has been shown to be associated in non-demented older adults with impairments in a variety of neuropsychological tests of learning, word fluency, visual-spatial abilities, and mental status. [62, 63]

Whilst hypothyroidism has classically been described as a cause of ‘reversible dementia’, there is a lack of evidence to show that there is complete resolution of neurocognitive deficits following treatment of hypothyroidism. [64] Further research is required to elucidate the potential role of perturbations of thyroid function as a contributor to a dementing process, as many of the studies to date have not been able to adequately address that hypothyroidism, co-morbidity, polypharmacy and alternative causes of dementia are all common in the elderly, and that interpreting the potential interplay between these factors is complex. [65]

In subclinical hypothyroidism, a number of studies have shown association with adverse cognitive function in younger individuals, [66–68] but results in older people have been conflicting. One study in individuals with a mean age of 74 years showed that people with subclinical hypothyroidism had worse performance on verbal recall and cognitive scores but working memory and processing speed were unaffected. [69] The PAQUID survey of individuals aged 65 years or more showed that increased TSH levels were significantly linked with the presence of symptoms of depression but not with impairment of cognitive function. [70] There have been other studies which do not support any association between subclinical hypothyroidism and cognitive impairment. [20, 62, 71, 72] The InCHIANTI study found a significant association between subclinical hyperthyroidism and cognitive impairment as assessed by the mini-mental state examination, but no such association with subclinical hypothyroidism. [12] A prospective observational study within the Leiden 85+ cohort, which followed up a total of 599 patients from the age of 85 to 89 years for a mean period of 3.7 years, found no significant association between thyroid dysfunction and either depression or cognitive impairment. This was a large and appropriately powered study, and the authors argue that in this very elderly cohort, whilst depression, dementia and thyroid dysfunction are all relatively common, the relationship appears coincidental rather than causal. [16] In contrast, in a notably younger cohort of predominantly euthyroid patients aged 49–71 years, a higher TSH level was associated with poorer performance in tests of memory. [19] Amongst the many as-yet unanswered questions about the relationship between thyroid status and cognitive ability, includes the possibility that the nature of this relationship changes with the aging process.

Studies which have addressed whether cognitive functioning improves with levothyroxine therapy in the context of subclinical hypothyroidism have returned conflicting results. Two small randomised controlled trials, recruiting between 19 and 37 patients respectively, have reported improvements in cognitive function with thyroid replacement therapy in subclinical hypothyroidism. [73, 74] However, two larger randomised controlled trials, including the Birmingham elderly thyroid study which enrolled 94 patients over the age of 65 years, showed no improvement with therapy. [49, 75]

#### Mobility and frailty

Higher TSH levels and subclinical hypothyroidism have been associated with a variety of improved health outcomes in the elderly population, including in domains pertinent to the considerations of mobility and frailty. Amongst the very elderly, there is evidence from the Newcastle 85+ study that lower TSH levels correlate with an increasing burden of nonthyroidal disease and disability. [18] Indeed, there is evidence from the Health ABC study suggestive of health benefits for older patients whose TSH levels lie within the subclinical hypothyroid range, as in this elderly cohort (mean age 75 years), those with a TSH (4.5–6.99 mU/L) had faster gait speed and superior cardiorespiratory fitness than those with lower TSH levels. [76] The Leiden 85+ study found no relationship between the serum TSH or fT4 and limitations to activities of daily living in people over the age of 85 years. [16] In the Zoetermeer study, a longitudinal population study of independent ambulatory predominantly euthyroid men, lower T4 and T3 levels were associated with improved physical functional status. [19] Quality of life assessment scores have been shown not to differ between the euthyroid and subclinical hypothyroid groups in an elderly population. [49]

There is relatively scant evidence on the effects of subclinical hypothyroidism on bone health. A Japanese study which employed quantitative heel ultrasound reported that although subclinical hypothyroidism does not appear to affect bone turnover there was an observed impact on bone structure. [77] The MrOS study found no association between TSH and bone loss as measured by sequential hip dual-energy X-ray absorptiometry, nor an increased fracture risk in the subclinical hypothyroid or hypothyroid categories. [78] An American prospective observational study which followed up a cohort of 3567 people over the age of 65 years for a median duration of 13 years found that subclinical hypothyroidism was significantly associated with increased risk of hip fracture in men with a hazard ratio of 2.31 (95% CI, 1.25–4.27) but not women. [79]

In summary, there is little evidence in the literature to clearly link overt hypothyroidism with reduced mobility or increased frailty, and in subclinical hypothyroidism there is some published data suggestive of improvements in these domains compared to euthyroid individuals, although the data is conflicting here.

#### Longevity

As the proportion of older people worldwide is increasing rapidly, the factors associated with healthy ageing have become the focus of intense research. Several theories have connected ageing with energy metabolism. One such proposed mechanism relates the lifespan of an organism with its size due to variation in resting metabolic rate. Another theory proposes that increases in free radicals that are generated due to oxidative metabolism are associated with the negative effects of ageing. [80] Thyroid hormones, via its effects on metabolism and the oxidative stress pathways, play a crucial role in the process of ageing and longevity. Experimental data effectively demonstrate the correlation between thyroid hormones and lifespan. Animal models of longevity, either naturally long-living or genetically modified, demonstrate low thyroid hormone. [81, 82] Age-related mild hypofunction of the thyroid gland seems to confer a longevity benefit. Numerous population-based studies have shown either a survival benefit, [16, 19, 83–85] or no adverse impact of lower thyroid function. [18]

As thyroid hormones have a direct impact on the metabolic rate of an individual and can thus play a key role in modulating longevity it is possible that thyroid hormone replacement in older hypothyroid individuals needs to be tailored differently to that of younger patients. However, the target TSH and thyroid hormone levels are uniform across the age groups and age-specific ranges are not utilised. This is despite the fact that the oldest age groups comprise the largest proportion of all hypothyroid. [3] Furthermore, over-treatment with thyroid hormones is common in older women, which is a risk factor for atrial fibrillation and osteoporosis. [86] In the United Kingdom, areas with higher levothyroxine prescribing are independently associated with atrial fibrillation. [87] No definitive trial of levothyroxine treatment in elderly hypothyroid patients comparing different TSH target values is currently available. A feasibility trial of manipulating thyroid hormone doses in older hypothyroid patients aiming for a higher serum TSH level concluded that a definitive trial is viable. [41]

### Treatment of hypothyroidism in the elderly

Despite the high prevalence of hypothyroidism, there have only been a few RCTs that have investigated outcomes with levothyroxine replacement. Three small RCTs in middle aged individuals with subclinical hypothyroidism showed improvement in cognitive function with levothyroxine replacement therapy. [73, 74, 88] Two larger RCTs with longer follow up have not shown any benefit in cognition with levothyroxine replacement, with the latter study specifically being in the elderly population aged 65 years or over. [49, 75]

The dose of levothyroxine that normalises serum TSH level is lower in older patients due to changes in thyroxine turnover with age related reduction in lean body mass. [89] Other factors such as decreased absorption, concomitant medication use, and other comorbidities could also affect thyroid hormone metabolism. The elderly are more susceptible to the ill-effects of thyroid hormone excess such as AF, [90] and osteoporotic fractures. [91, 92] Therefore, careful adjustments of levothyroxine dose at regular intervals are required in this population to avoid iatrogenic hyperthyroidism. The largest study to date of 12 months of levothyroxine treatment in subclinical hypothyroidism in older persons concluded that there was no benefit of treatment on quality of life or symptoms. [93] This double-blind, randomised, placebo-controlled trial of 737 patients older than 65 years with subclinical hypothyroidism demonstrated no significant improvement in the 100-point ThyPRO score (Thyroid-Related Quality of Life Patient-Reported Outcome) with low-dose levothyroxine treatment. [93] Therefore, in addition to the lower dose requirements related to thyroxine metabolism, based on the current evidence, it is reasonable to raise the target serum TSH up to 6 or 7 mU/L in persons greater than age 70–80 years particularly if they are at risk of cardiac arrhythmias or osteoporotic fractures.

## Conclusions

Thyroid hormones have an essential role in the functioning of nearly all tissues in the body at all stages. Thyroid function changes with age and these alterations are more pronounced at both ends of the life span. Current evidence suggests that a slight lowering of thyroid function in older individuals, as evidenced by a marginally raised serum TSH and low normal FT4, may not be associated with an adverse outcome and may, in fact, be beneficial. On the other hand, high thyroid function, as evidenced by a low TSH level needs careful monitoring and treatment considered if there is evidence of end-organ damage (such as osteoporosis or AF), or if serum TSH is suppressed. Despite major advances in our understanding of thyroid function and ecology, mainly due to improvements in assay techniques and high-quality epidemiological studies, several unresolved issues remain. It is currently unclear what the precise underlying mechanisms are behind the changes in thyroid function that are observed in older individuals. Moreover, it is uncertain whether these changes are part of healthy aging or are a bio-marker of underlying disease.

More research is required to fully understand why thyroid function changes in older individuals and whether modulation of thyroid hormones is advantageous for healthy aging and longevity. Mild thyroid hormone deficiency (or subclinical hypothyroidism) is more common in the elderly. But, if it is ‘normal’ and indeed desirable to have a slightly low thyroid function in older people then the current use of uniform reference ranges across all adult ages may need to be revised. Age-specific reference ranges may be required to diagnose thyroid disease with special reference to subclinical thyroid disease as well as to target serum TSH in patients on thyroid hormone replacement. And, in the future, it is possible that manipulation of thyroid function for health and longevity may be routinely practiced.

## Abbreviations

FT3: free thyroxine
FT3: free tri-iodothyronine
ROC: receiver operating characteristic
rT3: reverse tri-iodothyronine
T3: tri-iodothyronine (T3)
T4: thyroxine
TgAb: Thyroglobulin auto-antibody
TPOAb: thyroid peroxidase autoantibody
TSH: thyrotropin
